# Histopathological patterns of endometrial carcinoma in a tertiary hospital in North-West Nigeria

**DOI:** 10.3332/ecancer.2024.1651

**Published:** 2024-01-05

**Authors:** Olaniyi A Olatunde, Modupeola O Samaila, Mohammed I Imam, Kasiemobi E Uchime, Suleiman E Dauda

**Affiliations:** 1 Department of Anatomic Pathology and Forensic Medicine, Faculty of Basic Clinical Sciences, College of Medicine, Afe Babalola University, Ado-Ekiti 360231, Ekiti State, Nigeria; 2 Department of Pathology, Ahmadu Bello University Teaching Hospital, SHIKA, Zaria 810107, Kaduna State, Nigeria; 3 Department of Pathology, Aminu Kano Teaching Hospital, Kano 700233, Kano State, Nigeria; 4 Department of Histopathology, College of Medical Sciences, Abubakar Tafawa Balewa University, Bauchi 740272, Nigeria

**Keywords:** endometrial carcinoma, histology, morphology, Kano, Nigeria

## Abstract

**Background:**

There are relatively few studies in Nigeria, and indeed, sub-Saharan Africa that have documented the relative frequencies and histomorphological patterns of endometrial carcinoma. This study aimed to determine the relative frequencies and clinic-epidemiological characteristics of endometrial carcinoma and its histological variants in Kano, North-Western, Nigeria.

**Method:**

A 10-year retrospective study of all endometrial carcinoma cases in the Department of Pathology, Aminu Kano Teaching Hospital, Kano. All relevant information was retrieved and data was analysed using Statistical Package for Social Sciences version 22.

**Results:**

Endometrial carcinoma showed an increment in prevalence from 0.5% of all gynaecologic admission in 2008 to 1.0% in 2017. Type I endometrial carcinoma, specifically endometrioid adenocarcinoma accounted for 80% of cases, while endometrial serous carcinoma was the most common type II endometrial carcinoma representing 20% of cases. Over 75% of endometrial carcinomas occurred in postmenopausal women with a mean age of 59 years.

**Conclusion:**

There is a rise in the prevalence of endometrial carcinoma and endometrioid adenocarcinoma is the most common histologic type.

## Introduction

Worldwide, carcinoma of the endometrium is the sixth commonest diagnosed cancer of the female genital malignancy with an estimated 417,000 new cases and 97,000 deaths as of 2020 [1]. It is the most commonly diagnosed female gynaecologic malignancy in developed countries such as the United States of America, Europe and Canada [1].

The incidence rates vary worldwide, however, it is said to be ten times higher in developed countries like North America, Europe and Australia than in Africa and South Central Asia. The statistics by Global Cancer Observatory (GLOBOCAN) have shown a steady global increment in endometrial carcinoma from incident cases of 287,000–320,000 in the year 2015 to 382,069 in the year 2018 and 417,000 in year 2020 [2–5]. In sub-Saharan Africa, endometrial carcinoma is the third commonest female genital tract malignancy after cervical cancer and ovarian cancer with a prevalence that ranges between 4% and 11% of gynaecologic admissions diagnosed as endometrial cancer in Nigeria as against North Africa where it is the commonest gynaecological malignancy [6, 7].

Endometrial carcinoma is a disease of postmenopausal women in the 6th and 7th decade of life with an age incidence between 55 and 70 years and a mean age of 60 years in developed countries such as the United Kingdom and The United States of America, compared to lower figure of 56 years reported in developing countries such as Ghana and Nigeria respectively [9]. However, despite the fact that endometrial carcinoma occurs commonly in menopause, the majority of about 15% of endometrial carcinoma cases occur in the premenopausal period, of these 5% occur before 40 years of age. Endometrial carcinoma presents commonly as abnormal uterine bleeding with or without discharge, this is an unusual presentation in Nigeria where the advanced stage of the disease such as ascites, rectal bleeding, constipation, urinary bleeding, and hemoptysis is seen. Most cases of endometrial carcinoma present in the early stages of the disease with a resultant good prognosis and higher survival rate if early diagnosis and effective therapy are achieved [1, 5, 8, 9]. 

The aetiology of endometrial carcinoma remains unknown, however, associated predisposing risk factors have been attributed to chronic unopposed oestrogen stimulation either endogenously or exogenously. These include obesity, hypertension, nulliparity, infertility, unopposed oestrogen stimulation, early menarche and late menopause, tamoxifen long-time use in breast cancer, oestrogen replacement therapy, diabetes mellitus, history of ovarian or breast cancer, previous pelvic radiotherapy, long-term use of oral contraceptives, Stein-Leventhal syndrome, Turner syndrome, and Lynch syndrome [8–11]. 

Endometrial carcinoma arises from the inner lining of the uterus and is epithelial in origin. Over 30 years ago, Bokhman proposed two pathogenetic types of endometrial carcinoma with different signalling pathways. Type I endometrial carcinoma is the most common (70%–80%) type and is usually a low-grade, endometrioid type, oestrogen-dependent tumour, seen more in obese, nulliparous and post-menopausal women. They are due to chronic unopposed oestrogen stimulation of the endometrium, often related to endometrial hyperplasia and are ER+, PR+ and P53- usually with low Ki-67 expressivity. They are often localised tumours with favourable prognoses. Type II endometrial carcinoma (20%–30%), are high-grade, non-endometrioid tumour, non-oestrogen dependent tumour, seen in older women with background endometrial atrophy. They are ER-, PR- and P53+ with high Ki-67 expressivity and have the tendency for metastasis with poor prognosis. The type II tumours include serous papillary adenocarcinoma, mucinous adenocarcinoma, clear cell adenocarcinoma, and others [8, 12, 13]. 

Globally, there is no recommended standard screening guideline for endometrial carcinoma, however, management of the risk factors could be preventive. The guideline for diagnosing endometrial carcinoma has recommended the use of transvaginal ultrasonography or endometrial biopsy. Transvaginal ultrasonography is the first choice of study because it is available with high sensitivity, and inexpensive. However, endometrial biopsy is for definitive diagnosis. Other diagnostic tools readily available include hysteroscopy and magnetic resonance imaging for assessing the endometrial thickness and or structural abnormalities in the uterus. The availability of these modern imaging techniques as well as trained personnel has significantly improved and aided the diagnosis of endometrial carcinoma most especially in developing countries [14].

The mainstay of treatment is surgery (Total hysterectomy either as vaginal hysterectomy, total abdominal hysterectomy with or without bilateral salpingo-oophorectomy and pelvic and para-aortic lymphadenectomy, radical hysterectomy and total laparoscopic hysterectomy). These are done alongside pelvic washing in staging endometrial carcinoma. The International Federation of Gynecology and Obstetrics (FIGO) tumour-node-metastasis staging system of 2009 has stratified endometrial carcinoma into four stages (Stage I–IV). Stage I is tumour limited to the uterus. Stage II is tumour extending to the cervix but not outside the uterus. Stage II is tumour metastasis to the vaginal or parametrium as well as regional and para-aortic lymph nodes. Stage IV is intrabdominal or extra-abdominal metastasis. The combination of radiotherapy with surgery or radiotherapy alone is allowed for those not fit for surgery. The place of surgery combined with adjuvant chemotherapy either with cytotoxic drugs or hormonal therapy is recommended for those with metastasis. The usual presenting complaint for endometrial carcinoma is vaginal bleeding with or without discharges thus necessitating early diagnosis with good prognosis. The prognostic factors for endometrial carcinoma include age, race, tumour grade, tumour stage, oestrogen receptor status and histologic type. The risk of recurrence is about 10% beyond 5 years in endometrial carcinoma thus the need for long-term follow-up. Stage I and II endometrial carcinoma has favourable prognosis, as against the worst outcome for stage II and IV patient [14, 15].

There is growing concern about the increasing incidence of endometrial carcinoma especially in developing countries which may not be unrelated to lifestyle changes by Africans to the Western world, increased availability of diagnostic facilities, increased exposure to associated risk factors especially in the younger age groups with obesity, physical inactivity and in part increasing long life span in women, as well as increasing population of perimenopausal/menopausal women with low parity [5]. 

The concerted renewed research focus on endometrial carcinoma especially in developed countries is premised on the increasing incidence of the disease, modern drive for precision focus treatment and the increasing mortality rate of 1.9% per year average due to obesity [12, 16]. However, the paucity of studies in Nigeria literature documenting the general morphological pattern of endometrial carcinoma as well as knowledge of the disease burden is still abysmally low thus the aim of this study [16, 17]. This is more compiling especially in low-income resource countries like Nigeria where modern and advanced technological tools in disease diagnosis are not readily available and accessible to the general populace thus the use of morphologic features is still more reliable in patient disease management, especially in endometrial carcinoma [17, 18].

In sub-Saharan Africa, studies about the prevalence of endometrial cancer are few either because of inefficient or non-functional cancer registries or the fact that more studies are focused on the more common gynaecological malignancies of the cervix and ovary. Studies have reported poor prognosis in endometrial carcinoma most especially in the Black race [8, 9, 19].

This study was undertaken to characterise the prevalent rate, and age distribution and to analyse the histomorphologic variants of endometrial carcinoma in Aminu Kano Teaching Hospital, Kano, Nigeria.

## Methods

### Design of the study

This is a retrospective study undertaken to review the histopathologic pattern of all endometrial carcinoma from the 1st of January 2008 to the 31st of December 2017 using hematoxylin and eosin-stained histology slides of cases retrieved from the departmental records, bench book, and patient case folder.

### The setting

The study was carried out at the Department of Pathology, Aminu Kano Teaching Hospital, Kano, Kano State from 1^st^ January 2008 to 31st December 2017.

### Data collection

The patients' data (socio-demographic and clinical features) such as age, parity, type of cancer, clinical history, and histologic diagnosis were extracted from the departmental records, bench books, patient case folder and histology requisition forms using a data extraction form. Patients who had histologically confirmed endometrial carcinoma had their slides and tissue blocks extracted. These confirmed cases had new slides cut from the paraffin-embedded tissue blocks and stained routinely with haematoxylin and eosin. All cases of missing or faded slides had new sections and slides made for histologic diagnosis. These slides were all histologically re-examined and diagnosis made by an independent pathologist.

The histologic classification and the clinical staging using the FIGO criteria was done using the World Health Organisation (WHO) Classification of Tumour Pathology and Genetics Tumour of The Breast and Female Genital Organs 2016 [20].

### Inclusion and exclusion criteria

The inclusion criteria included all diagnosed cases of endometrial carcinoma during the study period. Excluded from this study were cases where both slides and blocks could not be found or inadequate clinical details.

### Data analysis

The analysis of the data was presented in proportions, frequency tables, and figures using the Statistical Package for Social Sciences version 22. The results obtained were presented in tables, bar charts, figures, relative frequencies as well and group percentages.

### Ethical approval

The ethical clearance was obtained from Aminu Kano Teaching Hospital Ethical Research Committee, the ethical clearance number (NHREC/21/08/2008/AKTH/EC/2595).

## Results

During the 10-year study period, 6465 gynaecological biopsy samples were analysed. Out of these, 670 (10.3%) were genital tract malignancies. Eighty-five of the genital tract malignancies were diagnosed histologically as endometrial carcinoma accounting for 12.6% of cases and an observed rise in the prevalence rate from 0.5% in 2008 to 1.0% in 2017 (Table 1). 

**Figure 1.**
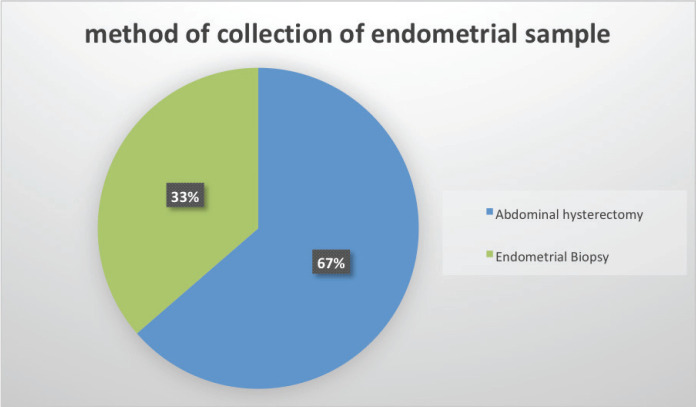
Method of endometrial sample collection.

Out of the 85 endometrial carcinoma cases, 55 cases met the inclusion criteria. These include 37 hysterectomy specimens and 18 endometrial biopsies (Figure 1).

**Figure 2.**
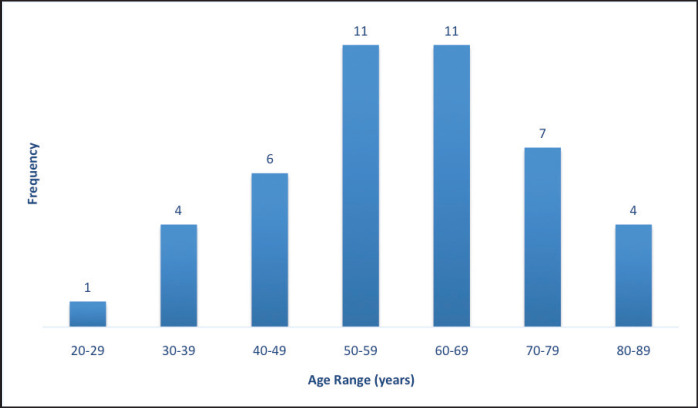
Age distribution of endometrial sample patients.

The age distribution ranged from 20 to 86 years with a mean of 59 years. The age distribution for type I endometrial carcinoma had two peaks in the 6th (50–59 years) and 7th (60–69 years) decades of life with 11 cases (25%), respectively. The peak age distribution for type II endometrial carcinoma was a decade later 8th (70–79) decades of life with 3 cases (Figure 2).

**Figure 3.**
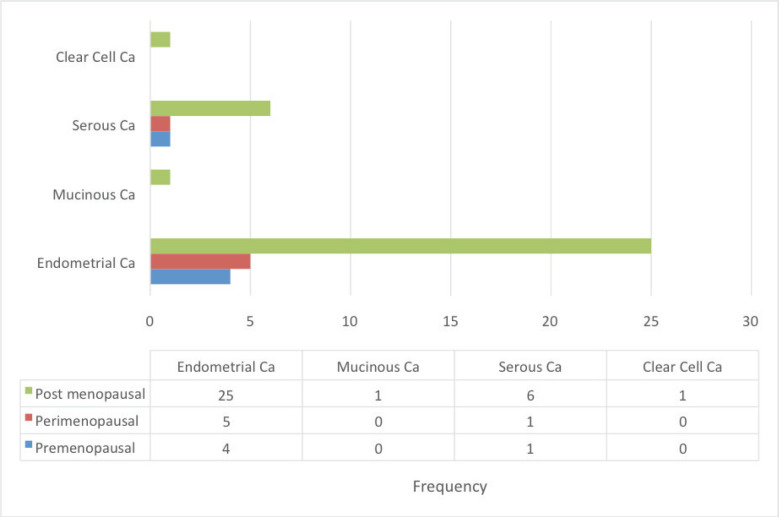
Endometrial carcinoma classification by menstrual age using 2016 WHO classification.

About two-thirds of cases were in the post-menopausal age range above 50 years while less than one-third were in the pre-menopausal age range less than 40 years old (Figure 3).

**Figure 4.**
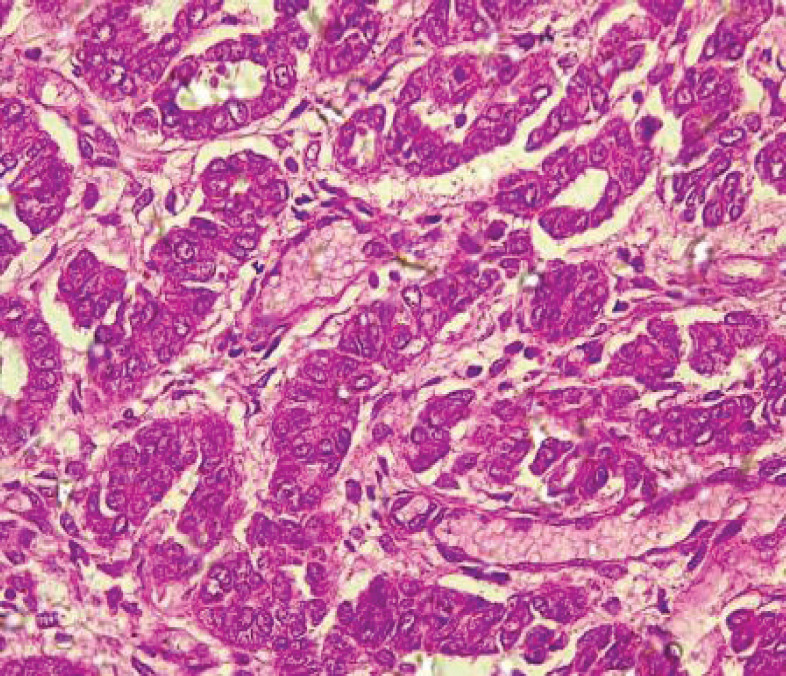
Endometrioid adenocarcinoma (type 1) showing malignant glands lined by simple to stratified columnar cells exhibiting pleomorphic nuclei with eosinophilic cytoplasm (H&E × 40).

**Figure 5.**
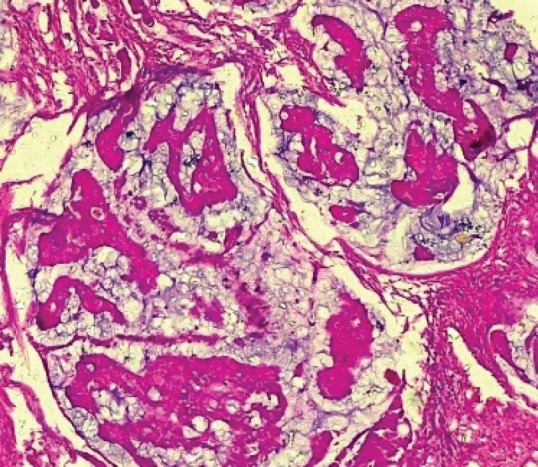
Mucinous carcinoma (type 1) showing malignant glands lined by simple to stratified columnar cells and disposed within extracellular mucin pools (H&E × 40).

Table 2 shows the 2016 WHO classification of all the analysed endometrial carcinoma cases using age distribution. Type I endometrioid carcinoma accounted for a greater proportion with 35 cases (79.5%) comprising endometrioid carcinoma and mucinous carcinoma variants. The endometrioid variant is morphologically characterised by well-formed glands with some villoglandular foci, lined by simple to stratified columnar cells, pleomorphic nuclei, and eosinophilic cytoplasm while mucinous carcinoma variants were characterised by columnar cells with basally located nuclei and mucin-rich cytoplasm (Figures 4 and 5).

**Figure 6.**
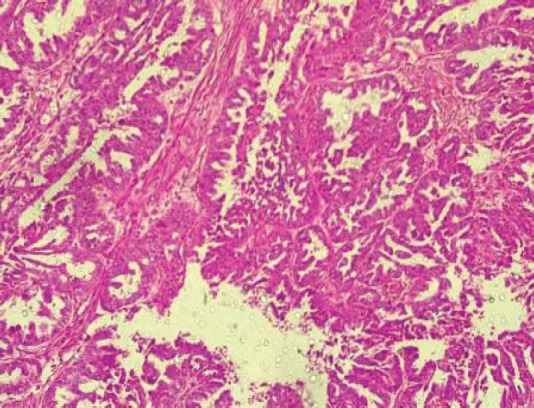
Serous carcinoma (type II) showing complex papillae with fibrovascular core (H&E × 40).

**Figure 7.**
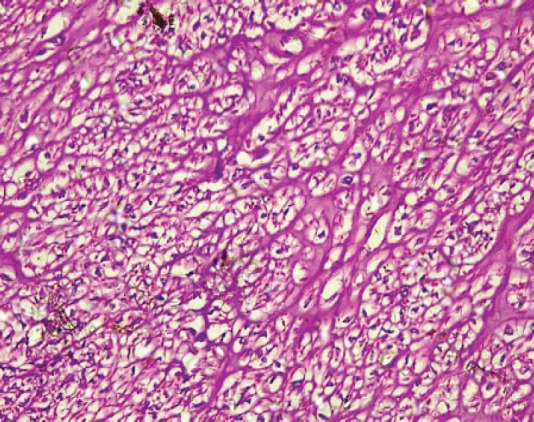
Clear cell carcinoma (type II) showing sheets of polygonal malignant cell with clear cytoplasm separated by thin fibrous band (H&E × 40).

The type II endometrial carcinoma made up of serous carcinoma and clear cell carcinoma variants accounted for 9 cases (20.5%). The serous variant was morphologically characterised by complex papillae with a fibrovascular core lined by vesicular nuclei with prominent eosinophilic nucleoli while the clear cell variant is composed of sheets of polygonal cells with clear cytoplasm separated by thin fibrous band (Figures 6 and 7).

**Figure 8.**
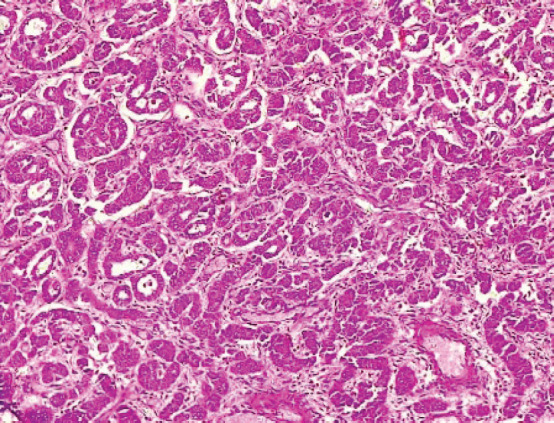
Grade I endometrioid carcinoma (type 1) showing glandular pattern of growth with less than 5% solid pattern (H&E × 40).

**Figure 9.**
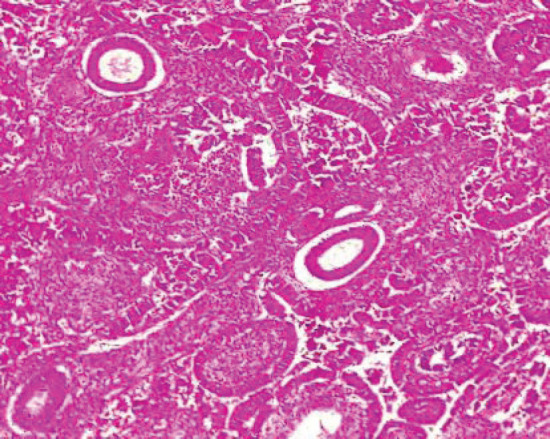
Grade II endometrioid adenocarcinoma (type 1) characterised by 6%–50% solid pattern with pleomorphic nuclei (H&E × 40).

**Figure 10.**
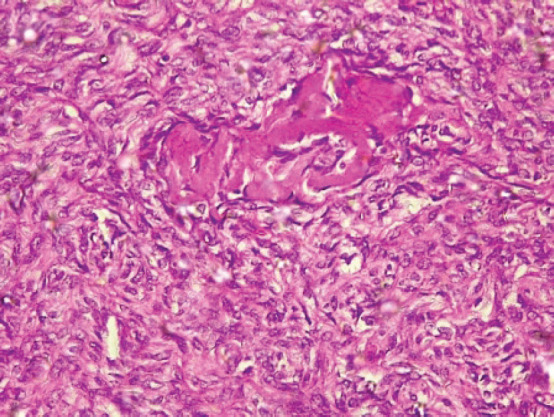
Grade III endometrioid adenocarcinoma (type 1) characterised by greater than 50% solid pattern with mitotic activity (H&E × 40).

The type I endometrial carcinoma was graded using the 2016 WHO protocol based on architecture and nuclear features. Grade 1 (well differentiated) accounts for 19 (56%) cases characterised by easily recognised well-formed glands histologically (Figure 8, Table 3). Grade II (Moderately differentiated) accounting for ten (29%) cases characterised by a mixture of well-formed glands with solid sheets of malignant cells (Figure 9). Grade III (Poorly differentiated) accounting for five (15%) cases, characterised by solid sheets of malignant cells with marked nuclei atypia and mitotic activity (Figure 10).

## Discussion

Endometrial carcinoma is the third most common female genital tract malignancy in sub-Saharan Africa. A recent study in Nigeria shows a prevalence rate of 4%–11% for all genital tract cancers [5]. Endometrial carcinoma in this study shows a prevalence of 12.6%. This is comparable to study in the same centre by Yakassai *et al* [21] (11.5%), Sanni *et al* [22] (13%) in Jos, North-Central Nigeria, Usman *et al* [23] (10.2%) in Maiduguri, North-East Nigeria, Joseph *et al* [24] (10.1%) in Abakaliki, South-East Nigeria and Seleye-Fubara and Uzoigwe [25] (13.3%) in Portharcourt, South-South Nigeria. The rising incidence rate from our study thus confirms the suggestion of the increasing incidence of endometrial carcinoma due to improved diagnostic techniques through the use of histopathology laboratory services and imaging techniques such as ultrasonography with available trained personnel as well as increased public awareness [5, 21]. However, this figure is higher than reports by Muhammad *et al* [5] (5%) in Zaria, North-west Nigeria, Okunowo *et al* [9] (1.4%) in Lagos, South-west Nigeria, Oriji *et al* [15] (0.68%) in Yenagoa, South-South Nigeria.

However, studies from other African countries show similar findings such as Meye *et al* [30] (5.3%) in Gabon, Kasule [31] *et al* (8%) in Zimbabwe, and Nkyekyer [28] (7.4%) in Ghana. This has been attributed to several African studies linking the low frequency of endometrial carcinoma to the incidence of endometrial hyperplasia without atypia with a 1% risk of malignant transformation to endometrial carcinoma [5]. GLOBOCAN 2012 report shows a similarly low figure of 2% in many Asian countries, which is in sharp contrast to the highest incidence rate of 12.7%–13.4% and 12.8%–15.6% reported in developed countries such as North America and North and western Europe [2, 26, 27].

This study further shows an incremental rise in the prevalence and burden of endometrial carcinoma as suggested by the rising trend in the incidence rate and the annual prevalence of endometrial carcinoma which may not be unrelated to associated risk factors. This is of concern as many low-income resource countries like Nigeria lack appropriate and dependable screening programs to prevent a public health epidemic of endometrial carcinoma [9].

About 75% of all diagnosed women with endometrial carcinoma in this study were more than 50 years old with a mean age of 59 years. This is consistent with data in other parts of Nigeria such as Enugu (56 years), and Zaria (54 years), and other countries such as Ghana (59 years), India (59 years), and Gabon (59 years) [28, 29]. However, the mean age in our study is variant to observations in the United kingdom (83 years) and the United State of America (89 years). This could be attributed to racial differences and age as an important risk factor [5, 9, 30–33]. Endometrial carcinoma is a disease of post-menopausal women with about 75% of women in our study being post-menopausal and about 11.3% of women being pre-menopausal. This is incongruent with studies that report about 20% of women being pre-menopausal. This is however contrary to findings in Zaria, Nigeria where 33% of women with endometrial carcinoma were pre-menopausal [5, 9].

The type I endometrial carcinoma, specifically endometrioid adenocarcinoma (80%) is the commonest histologic subtype in our study. This is consistent with studies that have documented the commonest histologic type of endometrial carcinoma as endometrioid adenocarcinoma [32–34]. This is also in agreement with data from India showing 80% as compared to values as high as 90% in developed countries such as the United States of America and the United Kingdom. Some collaborative studies among black women have shown a low prevalence rate for endometrioid endometrial cancer against a higher prevalence rate for non-endometrioid endometrial cancers [35–37].

The type II endometrial carcinoma accounted for 20% of the overall endometrial cancer in this study. This is consistent with findings in developed nations ranging from 6% to 20% for all endometrial cancers [34, 35, 38]. Serous papillary carcinoma and clear cell adenocarcinoma is the commonest histologic subtype of all type II endometrial cancers. They represent about 20% in this study of which serous papillary carcinoma is the most common with about 18.2%. Both serous papillary carcinoma and clear cell carcinoma are rare subtypes of endometrial carcinoma. They are high-grade tumours (stage III–IV) associated with poor prognosis and extremely high recurrence rate involving extra-pelvic sites such as the liver, lungs and upper abdomen. The survival rate is generally poor, however, when both subtypes are diagnosed as a stage I tumour, the prognosis is much better than in stage III. Both tumours respond poorly to treatment [14, 32, 34, 39–42].

## Conclusion

Our study has shown a rise in the prevalence rate for endometrial cancer. Endometrial carcinoma is more common in elderly women, the majority of whom are post-menopausal. The type I endometrioid carcinoma subtype accounted for about two-thirds of all endometrial cancer cases and the majority are in grade I with good prognosis. Serous papillary carcinoma accounted for about one-third of the type II endometrial carcinoma.

The global burden and increasing prevalence of cancer cases make it imperative for more concerted efforts to prevent a public health epidemic, especially in low-income countries like Nigeria. Presently much research funding focuses on cervical and ovarian cancer neglecting endometrial cancer. There has to be a predetermined concerted effort to drive research on the epidemiology and pathogenesis of endometrial cancer in other to improve the low level of public awareness of the disease burden and prevalence.

## Conflict of interest

There is no conflict of interest both financially and non-financially during this study

## Funding

There was no funding received for this study.

## Author contributions

Manuscript drafting and histopathologic reporting: DR Olaniyi A Olatunde, manuscript drafting, and histopathologic reporting: professor Modupeola O Samaila, Manuscript drafting, and histopathologic reporting: Dr Mohammed I Imam: revision of the manuscript revision: Dr Kasiemobi E Uchime, revision of manuscript: Dr Suleiman E Dauda. All authors approved the final version of the manuscript.

## Figures and Tables

**Table 1.** Shows the prevalence rate per year for endometrial carcinoma.

Years	Total endometrial samples per year	Total cases of gynaecologic malignancies per year	Total number of endometrial carcinoma per year	Number of endometrial carcinoma cases per year over total gynecologic malignancies per year(%)
2008	598	27	3	0.5
2009	671	72	9	1.3
2010	687	60	9	1.3
2011	727	84	11	1.5
2012	679	102	10	1.5
2013	785	67	10	1.3
2014	464	57	8	1.7
2015	533	69	6	1.1
2016	644	63	12	1.9
2017	677	64	7	1.0
Total	6,465	602	85	13.1

**Table 2.** Illustrate the age distribution of endometrial carcinoma cases diagnosed during the study period using the 2016 WHO classification.

Histologic type	Age								Total number	Percentage (%)
	<20	20–29	30–39	40–49	50–59	60–69	70–79	80–89		
Type I										
Endometrioid adenocarcinoma	0	1	3	5	9	9	3	4	34	77.2
Mucinous carcinoma	0	0	0	0	0	1	0	0	1	2.3
Type II										
Serous carcinoma	0	0	1	1	2	1	3	0	8	18.2
Clear cell carcinoma	0	0	0	0	0	0	1	0	1	2.3
Grand total	0	1	4	6	11	11	7	4	44	100

**Table 3.** Endometrioid adenocarcinoma (type I) grading (FIGO grading system) by WHO.

Grade	Number of cases	Percentage (%)
Grade I: less than 5% solid growth pattern without nuclear atypia	19	56
Grade II: 6% to 50% solid growth pattern with mild to moderate nuclear atypia	10	29
Grade III: more than 50% solid growth pattern with severe nuclear atypia	5	15
Total	34	100
